# The Relationship Between Cognition and Sensorimotor Behavior in an F1 Driving Simulation: An Explorative Study

**DOI:** 10.3389/fpsyg.2020.574847

**Published:** 2020-10-28

**Authors:** Nils Eckardt, Ingo Roden, Dietmar Grube, Jörg Schorer

**Affiliations:** ^1^Department of Sport and Movement Science, Institute of Sport Science, Carl von Ossietzky University of Oldenburg, Oldenburg, Germany; ^2^Department for Exercise & Health, Institute of Sport Science, Leibniz University Hannover, Hanover, Germany; ^3^Department of Educational Sciences, Institute of Social Science, Carl von Ossietzky University of Oldenburg, Oldenburg, Germany; ^4^Department of Educational Sciences, Institute of Education, University of Koblenz-Landau, Koblenz, Germany

**Keywords:** driving, cognition, esports, gaming, executive functions

## Abstract

Sensorimotor control simultaneously engages multiple cognitive processes, like decision making, intention, processing, and the integration of multisensory signals. The reciprocal relationship of cognition and sensorimotor learning is well documented. However, little is known if the status of cognitive skills relates to immediate sensorimotor performance of performing a novel skill. Thus, we aim to explore whether cognitive skills in general and executive functions (EFs) in particular may relate to novel sensorimotor performance and adaptive skills. Therefore, 23 male participants engaged in a novel driving simulation for 2 days. On the first day, they accustomed to the F1 simulation until meeting a preset threshold (adaption). On the second day, they aimed to drive as fast as possible (performance). In addition, we measured EFs and global cognition. We found meaningful relationships between response inhibition (Stroop Color and Word Test), the driving performance (*r* = 0.48, *p* = 0.013), and the adaptive ability (*r* = 0.34, *p* = 0.012). All other tests of executive functioning and global cognition remained non-significant. Our results illustrate an association of driving performance and adaptive abilities and the EF selective attention/inhibition in a novel F1 simulation. Given the novelty of the task, the ability to adjust sensorimotor behavior to keep the car on the track seems to be the primary necessary skill to navigate the lap and achieve fast times.

## Introduction

The importance of cognitive control processes becomes apparent especially when we engage in complex situation where multiple afferent signals from sensory inputs must be integrated and coordinate in an abundance of degrees of freedom (i.e., hand and feet or the whole body). In this article, we attempt to investigate how executive functions (EFs) may be related to immediate sensorimotor learning ability to a specific novel and complex sensorimotor skill (i.e., racing in an e-game).

The idea, that sensorimotor performance and aspects of cognition are interrelated, has gained wide acceptance nowadays (Seidler and Carson, 2017). Particularly of interest in this regard are EFs. EF comprises a family of cognitive processes associated with planning, organizing, and executing goal-directed behavior (Müller and Kerns, 2015). EFs help to regulate our behavior by governing afferent sensory information, aligning attention, and selecting a motor response (Diamond, 2013). Given the involvement of EF in motor planning, execution, and supervision, it does not surprise that components of EF (working memory, timing measures of inhibition, and set switching) correlate with sensorimotor performance (manual dexterity, ball skills, and balance) (Rigoli et al., 2012). While motor performance generally is operationalized using fine-motor coordination tasks like manual dexterity or balance performance, the relationship between EF and sensorimotor performance can be extended to multitasking skills like e-gaming. It is well documented that experienced e-gamer (mostly action game player) demonstrated increased performance in global cognition compared to non-players (see Hubert-Wallander et al., 2011 for a review). For example, Steenbergen et al. (2015) found that experienced action-game players outperformed non-players on a stop–change paradigm that provides a well-established diagnostic measure of action cascading. In addition, action-game players perform better at tests of EF, like task switching (Green and Bavelier, 2006), and tests of global cognition, like multiple object tracking (Cain et al., 2012) and dual-task performance (Strobach et al., 2012), among others.

Driving a car, also a virtual car, is a complex and “executive” task (Anguera et al., 2013). That is, car driving requires the coordination and a combination of multiple components (i.e., braking, accelerating, navigation, etc.) into a coherent global action. Based on our understanding of EF, which aligns with the framework by Miyake et al. (2000), a recent review by Walshe et al. (2017) stated that, out of the three core components of EF, working memory, inhibition, and set shifting, the latter was reported only in very few studies and none found a relationship with driving performance in a driving simulation. In contrast, inhibition, particularly in combination with working memory, responsible for governing multitasking, seems to be related to driving performance (Walshe et al., 2017). For example, Ross et al. (2015) showed that poor performance on inhibitory tasks and poor working memory together contributed to unsteady driving performance. Similarly, poorer inhibitory control on the Go/No–Go task positively correlated with risky behavior in a driving simulation (Hatfield et al., 2017). Similar, Mackenzie and Harris (2017) found that participants, who performed better at attentional functioning tests, exhibit more ecological eye movement and safer driving, like lane maintenance. However, a study by Van Leeuwen et al. (2017) showed that there are no major differences in a non-domain specific choice reaction time task and tracking task between racing and non-racing drivers. Not surprisingly, racing drivers drove faster laps than their controls. Naturally, this is due to years of sensorimotor learning. The authors showed that this was mainly because of more sensorimotor control in lane control and anticipatory gaze behavior. A recent investigation by Wood et al. (2016) showed that working memory capacity, measured with an automated version of the operation span task, correlates with hazard perception performance and self-reported driving behavior. The authors point out the relationship between controlled visual attention in motor-cognitive interference tasks and driving behavior, particularly in a more ecological environment like a driving simulation.

All above-mentioned studies showed that (driving) performance is not only based merely on superior sensorimotor behavior and intelligence but also on cognitive domain-specific sets, learned and adapted over years and years of specific practice (Lappi, 2015). However, little is known if different levels of cognitive skills, measured by tests of EF and global cognition, may relate to novel sensorimotor performance. Given the importance of EF in learning paradigms and the overall important role in driving performance, a relationship between cognition and sensorimotor behavior may impact study designs of any investigation regarding sensorimotor learning, at least in simulated driving studies. Therefore, the aim of the current study was to explore the relationship between EFs, global cognition, and sensorimotor behavior in a novel F1 driving simulation. We would hypothesize first that there is a relationship between fast driving, i.e., performance, and EF, based on the review by Walshe et al. (2017). Second, we would assume that global cognition does not relate to performance because of the inconsistent results throughout the literature. Given the novel nature of the sensorimotor task, we stratified participants in a short adaption session. Similar to performance, we would assume, third, that adaptive skills would relate to EF and, fourth, that there is no relationship between adaption and global cognition.

## Materials and Methods

### Participants

We performed a statistical power analysis for sample size estimation. The targeted effect size (ES) in this study was medium to large, based on several investigations (Ross et al., 2015; Hatfield et al., 2017), using Cohen’s criteria (Cohen, 1988). With an alpha of 0.05 and a power of 0.80, the projected sample size needed for this ES is approximately *N* = 23–67 for a bivariate normal correlation model (GPower 3.1.9.2). In total, 23 male participants (*M*_*age*_ = 25.6, SD = 2.2) participated voluntarily in this study. They were recruited by word-of-mouth advertising. Exclusion criteria were any neurological or musculoskeletal pathology, uncorrected visual impairment, and a professional occupation in e-gaming (competitive player who is paid to play video games). Our participants were engaged in e-gaming (*M*_*hours/week*_ = 1.1, SD = 0.9) but had no previous experienced in a driving simulation. On average, participants received their driving license 8 years ago (*M*_*years*_ = 8, SD = 2). The participants where naive to the study hypothesis. All participants provided written informed consent prior to enrollment. The local ethics committee of the University of Oldenburg gave their approval (EK/2020/049), and we complied with the relevant ethical standards of the latest Declaration of Helsinki (WMA, October 2013).

### Apparatus

The driving evaluation was conducted with an F1 simulator (The Codemasters Software Company Limited). The simulation was run on a PC, using Windows 10 (i7-8700 CPU @ 3.20 GHz, NVIDIA GeForce GTX 1070 graphics card). The visual system consisted of a 27-in screen (BenQ XL2720T), with a resolution of 1,920 × 1,080 pixels, a refresh rate of 120 Hz and a latency of 1 ms. Auditory feedback was provided through external speakers set to ∼60 dB for all participants. To pilot the simulation, we used a steering wheel (ClubSport wheel Formula Carbon, Fanatec^®^, Landshut, Germany), throttle, and brake pedals (CSL Elite Pedale LC, Fanatec^®^, Landshut, Germany). The steering wheel was mounted on wheel-base pedals (ClubSport Wheel Base V2.5, Fanatec^®^, Landshut, Germany) providing force feedback. The driving aids *traction control* and *ABS* were fully enabled, while gear shifting was set to manual. All other driving aids were disabled. Participants were tested under the same conditions: track, Austria; car, Ferrari; perspective, third person; session, practice (without opponents).

### Measures of Executive Functions

Our understanding of EF aligns with the framework by Miyake et al. (2000), emphasizing (a) shifting between tasks or mental sets, (b) updating and monitoring of working memory representations, and (c) inhibition of dominant or prepotent responses. While there is evidence that these target EF are moderately correlated, they contribute differentially to performance on complex executive tasks. Given the indication of non-meaningful influence of set-shifting abilities for driving performances (Ross et al., 2015; Hatfield et al., 2017; Walshe et al., 2017), we choose not to include data about those abilities. In addition, tests comprising global cognitive functions were used in the present study. All computerized tests of EF where based on the psyToolkit (Stoet, 2017) but translated to German. We recorded the computerized tests using a Macbook Pro (Apple Inc.) with a 13.3″ screen, running macOS Sierra Version 10.12.6.

### Response Inhibition

#### Stroop Color and Word Test

To assess the ability to inhibit cognitive interferences and selective attention, we administered a computerized Stroop Color and Word Test (Penner et al., 2012). Forty color–word sets were shown in a random order on a computer screen, and participants had to recognize the color of the word by pressing a corresponding key on the keyboard. We presented 20 congruent word–color sets, where the color (i.e., red) and the word (i.e., red) of the presented stimuli were identical and 20 incongruent word–color sets, where the word (i.e., red) did not match the color (i.e., green). The ability to selectively attend and control response output was calculated as the time ratio (i.e., Stroop Score) of color–word interference and congruent tasks (incongruent/congruent).

#### Simon Task

Another test to assess selective attention and conflict resolution is the Simon Task (Hommel, 2011). In contrast to the Stroop Color and Word Test, participants are faced with a potential spatial conflict. The words “left” or “right” are presented either left or right of a central cross. Participants were asked to press “A” on the left side of the keyboard when they recognized the word “left” and press “L” on the right side of the keyboard when they recognized the word “right.” The ability to identify and control response output was calculated as the time ratio (i.e., Simon Score) of conflicting and non-conflicting spatial presentations (conflict/non-conflict).

### Visuospatial Memory

We used a computerized version of the Corsi Block Test to access visuospatial short-term working memory (Kessels et al., 2000). The task represents the participant’s ability to remember series of spatial locations presented in sequences of different lengths. Participants were asked to memorize and reproduce the sequence of two to nine squares displayed on a computer screen. At the beginning of the Corsi Block Test, a sequence of two squares appears for a short time of 250 ms. Immediately after the sequence, participants were asked to repeat the sequence correctly. If the reproduction was correct for two trials, the sequence was increased by one square. If participants failed to reproduce the correct sequence for two trials, or if they reached the maximum of nine squares, the test was over.

### Mental Rotation

In this task, 10 versions of triplets with random geometric forms were presented simultaneously. The triplets were presented in triangular, where the top form represented the original and the bottom forms were either rotated or mirrored (Hirschfeld et al., 2013). The task was to mentally rotate the top geometric form and decide which geometric form (A or B) was rotated and which was a mirrored image of the form on the top. Participants were instructed to press the A key (left image) or L key (right image) on a keyboard to indicate the rotated image.

### Working Memory Span

To test working memory, we used the Digit Memory Test. This test consists of two parts: A, Digit Span Forward and B, Digit Span Backward. The assessor read out a random sequence of numbers (one per second) aloud, beginning with three digits and ending with nine digits. Participants were asked to either recall the sequence in normal (Part A) or reverse order (Part B). Each correctly repeated sequence counted as 1 point. The total number of correct responses (backward and forward) were added and converted into a standard score, indexing working memory capacity (Turner, 2005).

### Procedure and Measures of Driving Performance

The testing was conducted over two consecutive days. An assistant who was trained in administering the computerized EF test battery ran the data collection. On day 1, participants received a paper handout explaining the experiment, and they had to sign an informed consent. Then, participants performed the EFs tests. After completing the tests of executive functioning and a 10-min break, participants took a seat in front of the desktop computer with the driving simulation. The assessor shortly explained the necessary functions of the driving simulation, such as throttle, breaks, steering, and shifting. Participants were instructed to drive the fastest possible lap time while keeping their vehicle on the road, i.e., they were not allowed to cut corners. If participants took a shortcut, like leaving the track, the simulation marked this as invalid lap and the assessor restarted the lap. All sessions begun with a flying start, just before the final curve heading on the home straight. However, the session on the first day did not serve the purpose of driving the overall fastest personal time but to merely parallelize participants skills and test their adaptive skills, given that this was a novel task for all participants. We ended the session if participants undercut the 1:20 min twice or after 30 min. The time until participants reached 1:20 min was recorded and operationalized adaptive skills (*Adaption*). The threshold of 1:20 min was chosen because it requires a minimum of control over the simulation and lane maintenance. During pilot testing, we felt that after 30 min of engaging in the simulation, we experienced some signs of mental fatigue and choose this threshold. On the second day, participants were instructed to drive the fastest possible lap time. After they undercut 1:20 min again, the time of 10 valid laps were recorded, averaged, and subjected for further analysis. The mean lap time served as *Driving Performance*.

### Statistical Analysis

We conducted an explorative cross-sectional study, examining the relationship between EFs and sensorimotor behavior in a driving simulation. First, we checked for normal distribution by determining the significance of skewness. Therefore, we calculate skewness *z*-scores by dividing them by their associated standard error. A *z*-score of ≥1.96 determines a significant skewness. We ran either Pearson’s rho Bayesian correlation analysis, or in case of violating the assumption of normality or ordinal scaled data, the non-parametric alternative Kendall’s rho instead. Further, we calculated BF_+0_, the Bayes factor that quantifies evidence for the one-sided alternative hypothesis that the population correlation is higher than 0, with a stretched beta prior width of 1. In addition, we report 95% credible intervals of the posterior density of the correlation (Wetzels and Wagenmakers, 2012). Alpha was set at 5%. All data were analyzed using the freeware JASP (Version 0.9.2).

## Results

Given that we recruited 23 participants, which is on the lower bound of the required number of participants due to the power analysis, we would discuss non-significant results extensively and only with caution.

The only variable showing significant skewness was “Adaption;” therefore, we used Kendall’s rho for further analysis for this variable. After the inspection of the Bayes Factor robustness check, we are confident that choosing a prior width of 1 was appropriate, given that the results would not change using different prior width.

Furthermore, we checked if driving experience (duration of having a driver’s license in years) has an effect on our variables of interest. However, we found that driving experience has neither effect on “Performance” (*r* = 0.15, *p* = 0.490) nor on “Adaption” (*r* = -0.27, *p* = 0.090).

**FIGURE 1:**
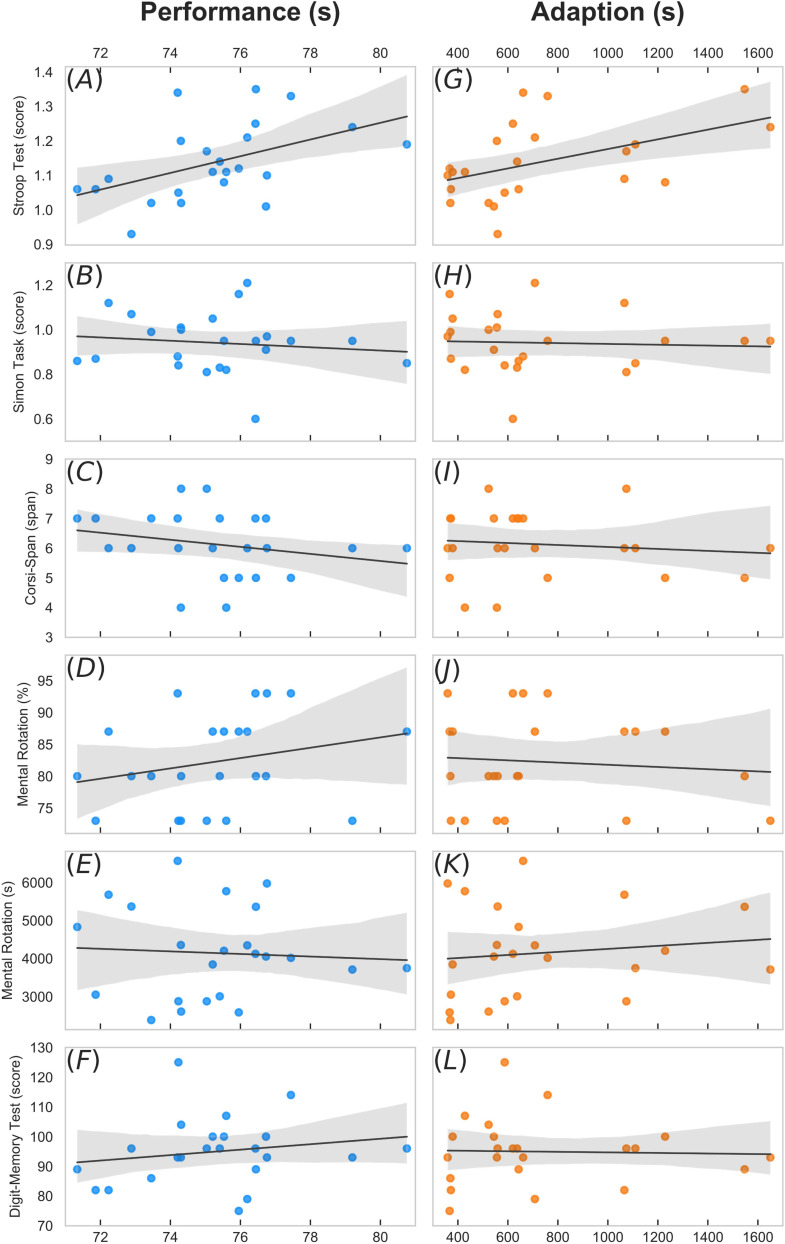
Results of correlation analyses performed. Scatter plots depicts Pearson’s correlations between **(A–F)** performance, **(G–L)** adaption and our variables of interest (executive functions and measures of global cognition). The straight line represents the linear regression model fit and the gray shade its 95% CI.

Descriptive results are shown in Table 1. All correlation results are visually presented in Figures 1A–L and in Table 2. Across all participants, the ability to selectively pay attention and inhibit response output measured by the Stroop Color and Word Test was positively related to driving performance (*r* = 0.48, *p* = 0.013; 1−β = 0.78, Figure 1A) and the ability to adapt quickly to a novel driving simulation task (*r* = 0.34, *p* = 0.012; 1−β = 0.49, Figure 1G). We found moderate evidence (Bayes Factor) for a relationship. The other measures of EF and global cognition remained without meaningful relationships.

**TABLE 1:** Descriptive values.

	**M**	**SD**	**95% CI**
Time (sensorimotor performance in s)	75.28	2.22	71.44, 80.45
Adaption in s	728.2	373.70	360, 1629
Trials to adaption (*n*)	22.7	7.8	13.2, 39.4
Driving experience (years)	8.0	2.3	5, 11
Time engaging in e-gaming (h/w)	1.46	0.92	0.96, 2.20
Stroop Score (incongruent/congruent)	1.14	0.11	0.95, 1.35
Simon Score (incongruent/congruent)	0.94	0.13	0.64, 1.20
Corsi Span Task (span)	6.13	1.10	4, 8
Mental rotation (% correct responses)	82.26	7.22	73, 93
Mental rotation (time per trial in s)	4.14	1.20	2.42, 6.45
Digit Memory Test (score)	94.96	11.14	75.8, 122.8

**TABLE 2:** Correlation outcomes.

	***r***	***r−*95% CI**	***p***	**BF_+0_**
**Sensorimotor performance (s)**				
Stroop Score (incongruent/congruent)	0.48	0.10, 0.72	0.013	6.47
Simon Score (incongruent/congruent)	–0.12	0.01, 0.38	0.771	0.18
Corsi Span Task (span)	–0.23	0.02, 0.43	0.168	0.07
Mental rotation (% correct responses)	0.25	0.02, 0.58	0.262	0.83
Mental rotation (time per trial in s)	–0.06	0.01, 0.41	0.289	0.21
Digit Memory Test (score)	0.18	0.01, 0.54	0.184	0.57
**Adaption (s)**				
Stroop Score (incongruent/congruent)	0.34	0.09, 0.72	0.012	6.30
Simon Score (incongruent/congruent)	–0.09	0.01, 0.27	0.592	0.18
Corsi Span Task (span)	–0.08	0.01, 0.28	0.695	0.18
Mental rotation (% correct responses)	–0.01	0.01, 0.32	0.658	0.28
Mental rotation (time)	0.12	0.01, 0.39	0.287	0.58
Digit Memory Test (score)	–0.08	0.01, 0.32	0.557	0.28

**df* = 21; BF_+0_, Bayes factor that quantifies evidence for the one-sided alternative hypothesis that the population correlation is higher than 0.*

Given different places on the track of lane violations, there was a heterogenous amount of trials to threshold (adaption). However, the number of trials until the threshold was reached was no mediating factor and had, thus, no relation to adaption time or performance.

## Discussion

Previous studies have demonstrated a relationship between EFs and sensorimotor performance in driving (Walshe et al., 2017). Here, we explored the relationship between EF and sensorimotor performance in a novel driving simulation task. All our hypotheses were supported. While global cognition and working memory reveal no relationship with neither driving performance nor adaptive ability, measures of EF such as selective attention and inhibitory behavior are meaningfully related to both driving performance and the time to adapt to a novel task.

The findings are in line with previous research, particularly the results of Ross et al. (2015), stating that the inhibition of inappropriate or non-ecological reactions are important for drivers to maintain a less variable traffic line. These inappropriate reactions might be unnecessary steering maneuvers happening when scanning for the next braking point and/or scanning the environment. This might result in a reduced time to adapt a novel task and to an increased driving performance (faster lap times). In other words, people with improved inhibition abilities would faster release the foot from the accelerator and might show faster braking times. Walshe et al. (2017) summarized that drivers with particularly high speed violation rates as well as drivers with low inhibition controls for speed could be associated with poorer inhibition skills. The authors assume that the poor inhibition control may contribute to the inability to ignore irrelevant information and therefore disturb speed control and general control on the track. The other domains of EF do not seem to be that important to driving behavior, although the authors of the above-mentioned review question the non-association of set shifting and driving, mostly due to the poor examination rate of set shifting in the literature (Walshe et al., 2017). In line with Van Leeuwen et al. (2017), we found no relationship between global cognition and performance. While they compared racing and non-racing drivers, the choice reaction time task and tracking task had no distinctive effect between the performance levels. The authors explain this with task-specific differences in sensorimotor skills between experts and novices while there are no major differences in general cognitive abilities. Yet, this is not directly comparable to our study, given that our participants were roughly at the same sensorimotor level. However, the small differences in response inhibition in our study, most likely leading to safer and more controlled driving, resulted in better performance.

In contrast to Wood et al. (2016), we did not find a relationship of performance and safe driving (lane maintenance) and working memory. Similar to set shifting, this EF seemed to be not important to our task. This does not exclude that, with a more complex task, we would see correlations between working memory and performance and adaption. Although the latent variables of EF do correlate with each other (Miyake et al., 2000), the correlation coefficient between updating/working memory and inhibition is moderate, meaning that task specificity may be responsible for the non-significant result in working memory and performance/adaption.

Interestingly, we found an effect for the Stroop Color and Word Test but not the Simon Task, although they are logically similar. Both produce two sources of interference in information processing; however, in recent years, it was debated whether the conflicts resulting from the two tasks are resolved by the same or different mechanisms (Scerrati et al., 2017). The authors assume that, in contrast to the Simon Task, stimuli in the Stroop Color and Word Test are unrelated to the response, and processing might be already occurring at the stimulus identification stage, rather than at the response level. In addition, the perceptual account claims that the interference of the Stroop Color Task, resulting from a semantic conflict (i.e., ink color vs. meaning of the word), and the interference in the Simon Task, stemming from a non-semantic conflict (i.e., different locations for stimulus and response) causes distinct conflicting effects (Li et al., 2014; Scerrati et al., 2017). Therefore, it is possible that, although both tasks are investigating response inhibition, the nature of the specific sensorimotor task (i.e., e-gaming) determines relevant effects rather for the Stroop Color and Word Test than for the Simon Task.

The current study has some limitations that warrant discussion. First, we included exclusively male participants. More studies are needed in order to verify the reliability and repeatability of our findings in samples with both sexes. Second, some might argue that we did not administer the full range of EF tests, like set shifting. However, prior research reported no significant influence of set-shifting abilities for driving performances (Fazeli et al., 2013; Hatfield et al., 2017; Walshe et al., 2017). For this purpose, we decide not to include any task for measuring set-shifting abilities. Third, we included 23 participants, which is at the lower bound of our estimated sample size, meaning that we might had some false negatives and missed certain existing relationships. Therefore, we tried to avoid extensive discussions on non-significant results.

## Conclusion

In summary, our results illustrate an association of driving performance and adaptive abilities and the EF selective attention/inhibition in a novel F1 simulation. Given the novelty of the task, the ability to adjust sensorimotor behavior to keep the car on the track seems to be the primary necessary skill to navigate the lap and achieve personal fastest times. We would expect that other components of EF such as set shifting and working memory would be more important in a learning paradigm when participants must remember the optimal acceleration and braking landmarks and may deal with opponents. Indeed, there is some evidence that the ability to maintain attention is related to skill learning in driving contexts (Drews et al., 2008). Furthermore, since there is lack of secondary tasks presented, which we would face in a common real-world driving situation like listing to music, talking, and being aware of the environment and other drivers/opponents, our driving situation was more “clinical.” In addition, participants did not have to interact with opponents, as they drove in a training session without any other vehicle on the lap. Apart from a minor rule (i.e., do not take a short cut→invalid lap), no sanctions were applied for misbehavior, bad driving, crossing the lane, etc. Thus, additional EFs, i.e., long-term memory and particularly set shifting, might play a vital role in other experimental setups. Previous research into action gaming indicated benefits of playing such games for memory (Li et al., 2015). Whether this assumption can be transferred to a driving simulation needs to be tested in future studies.

The results support the relationship of EF, sensorimotor performance, and learning. Our findings add further information on more ecologically valid research regarding sensorimotor learning. Given the importance of EF in learning paradigms and the important role in driving performance, we would suggest that studies that investigate sensorimotor learning may have to stratify their participants due to EF performance rather than randomize them.

## Data Availability Statement

The datasets presented in this study can be found in online repositories. The names of the repository/repositories and accession number(s) can be found in the article/ Supplementary Material.

## Ethics Statement

The studies involving human participants were reviewed and approved by the Kommission für Forschungsfolgenabschätzung und Ethik (EK/2020/049). The patients/participants provided their written informed consent to participate in this study.

## Author Contributions

NE analyzed the data, wrote the manuscript, and contributed to the conception and design of the study. IR, DG, and JS contributed to the study design, assisted with the data analysis and interpretation, and critically reviewed the manuscript. All authors read and approved the final version of the manuscript.

## Conflict of Interest

The authors declare that the research was conducted in the absence of any commercial or financial relationships that could be construed as a potential conflict of interest.
